# 3-Ethyl­sulfinyl-2-(4-fluoro­phen­yl)-5-iodo-7-methyl-1-benzofuran

**DOI:** 10.1107/S1600536810005581

**Published:** 2010-02-13

**Authors:** Hong Dae Choi, Pil Ja Seo, Byeng Wha Son, Uk Lee

**Affiliations:** a Department of Chemistry, Dongeui University, San 24 Kaya-dong Busanjin-gu, Busan 614-714, Republic of Korea; b Department of Chemistry, Pukyong National University, 599-1 Daeyeon 3-dong, Nam-gu, Busan 608-737, Republic of Korea

## Abstract

In the title compound, C_17_H_14_FIO_2_S, the 4-fluoro­phenyl ring is rotated out of the benzofuran plane, as indicated by the dihedral angle of 15.60 (6)°. The crystal structure exhibits an I⋯O inter­action [3.052 (2) Å].

## Related literature

For the crystal structures of similar 3-ethyl­sulfinyl-2-(4-fluoro­phen­yl)-5-halo-1-benzofuran derivatives, see: Choi *et al.* (2010**a* ▶,b* ▶). For the pharmacological activity of benzofuran compounds, see: Aslam *et al.* (2006 ▶); Galal *et al.* (2009 ▶); Khan *et al.* (2005 ▶). For natural products with benzofuran rings, see: Akgul & Anil (2003 ▶); Soekamto *et al.* (2003 ▶). For a review of halogen bonding, see: Politzer *et al.* (2007 ▶).
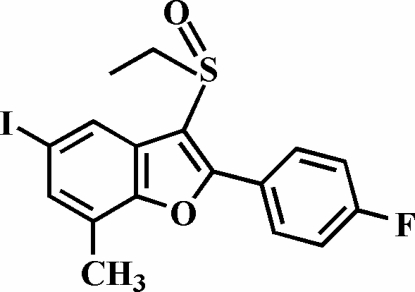


## Experimental

### 

#### Crystal data

C_17_H_14_FIO_2_S
*M* *_r_* = 428.24
Triclinic, 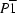

*a* = 7.4681 (5) Å
*b* = 10.3023 (7) Å
*c* = 10.9761 (7) Å
α = 76.289 (1)°
β = 89.138 (1)°
γ = 74.802 (1)°
*V* = 790.73 (9) Å^3^
*Z* = 2
Mo *K*α radiation
μ = 2.17 mm^−1^
*T* = 273 K
0.50 × 0.25 × 0.25 mm

#### Data collection

Bruker SMART APEXII CCD diffractometer
Absorption correction: multi-scan (*SADABS*; Bruker, 2009 ▶) *T*_min_ = 0.520, *T*_max_ = 0.582
6867 measured reflections
3386 independent reflections
3261 reflections with *I* > 2σ(*I*)
*R*_int_ = 0.049

#### Refinement

*R*[*F* ^2^ > 2σ(*F* ^2^)] = 0.022
*wR*(*F* ^2^) = 0.059
*S* = 1.06
3386 reflections
201 parameters
H-atom parameters constrained
Δρ_max_ = 0.71 e Å^−3^
Δρ_min_ = −0.81 e Å^−3^

### 

Data collection: *APEX2* (Bruker, 2009 ▶); cell refinement: *SAINT* (Bruker, 2009 ▶); data reduction: *SAINT*; program(s) used to solve structure: *SHELXS97* (Sheldrick, 2008 ▶); program(s) used to refine structure: *SHELXL97* (Sheldrick, 2008 ▶); molecular graphics: *ORTEP-3* (Farrugia, 1997 ▶) and *DIAMOND* (Brandenburg, 1998 ▶); software used to prepare material for publication: *SHELXL97*.

## Supplementary Material

Crystal structure: contains datablocks I. DOI: 10.1107/S1600536810005581/pb2023sup1.cif

Structure factors: contains datablocks I. DOI: 10.1107/S1600536810005581/pb2023Isup2.hkl

Additional supplementary materials:  crystallographic information; 3D view; checkCIF report

## Figures and Tables

**Fig. 1.**
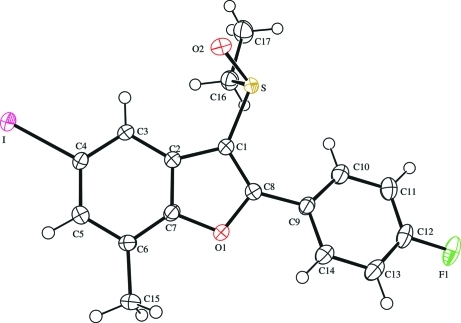
The molecular structure of the title compound with the atom numbering scheme. Displacement ellipsoids are drawn at the 50 % probability level. H atoms are presented as a small spheres of arbitrary radius.

**Fig. 2.**
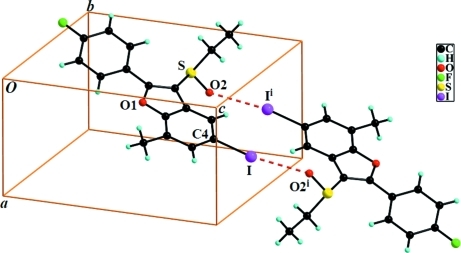
C—I···O interactions (dotted lines) in the crystal structure of the title compound. [Symmetry codes: i) - x + 1, - y + 1, - z + 2.]

## supplementary crystallographic information

### Comment

Compounds containing benzofuran skeleton show diverse pharmacological activities such as antifungal (Aslam *et al.*, 2006), antitumor and antiviral (Galal *et al.*, 2009), antimicrobial (Khan *et al.*, 2005) properties. These compounds widely occur in nature (Akgul & Anil, 2003; Soekamto *et al.*, 2003). As a part of our ongoing studies of the effect of side chain substituents on the solid state structures of 3-ethylsulfinyl-2-(4-fluorophenyl)-5-halo-1-benzofuran analogues (Choi *et al.*, 2010*a,b*), we report the crystal structure of the title compound (Fig. 1).

The benzofuran unit is essentially planar, with a mean deviation of 0.012 (2) Å from the least-squares plane defined by the nine constituent atoms. The dihedral angle formed by the benzofuran plane and the 4-fluorophenyl ring is 15.60 (6)°. The crystal packing (Fig. 2) is stabilized by an I···O halogen bond between the iodine and the oxygen of the S═O unit [C4—I···O2^i^ = 3.052 (2) Å; C4—I···O2^i^ = 168.75 (7)°] (Politzer *et al.*,  2007).

### Experimental

77% 3-Chloroperoxybenzoic acid (202 mg, 0.9 mmol) was added in small portions to a stirred solution of 3-ethylsulfanyl-2-(4-fluorophenyl)-5-iodo-7-methyl-1-benzofuran (330 mg, 0.8 mmol) in dichloromethane (25 mL) at 273 K. After being stirred at room temperature for 4h, the mixture was washed with saturated sodium bicarbonate solution and the organic layer was separated, dried over magnesium sulfate, filtered and concentrated at reduced pressure. The residue was purified by column chromatography (hexane–ethyl acetate, 2:1 v/v) to afford the title compound as a colorless solid [yield 82%, m.p. 466–467 K; *R*_f_ = 0.54 (hexane–ethyl acetate, 2:1 v/v)]. Single crystals suitable for X-ray diffraction were prepared by slow evaporation of a solution of the title compound in acetone at room temperature.

### Refinement

All H atoms were positioned geometrically and refined using a riding model, with C—H = 0.93 Å for aryl, 0.97 Å for methylene, and 0.96 Å for methyl H atoms. *U*_iso_(H) = 1.2*U*_eq_ (C) for aryl and methylene, and 1.5*U*_eq_(C) for methyl H atoms.

### Crystal data

**Table:**

C_17_H_14_FIO_2_S	*Z* = 2
*M**_r_* = 428.24	*F*(000) = 420
Triclinic, *P*1	*D*_x_ = 1.799 Mg m^−^^3^
Hall symbol: -P 1	Mo *K*α radiation, λ = 0.71073 Å
*a* = 7.4681 (5) Å	Cell parameters from 6089 reflections
*b* = 10.3023 (7) Å	θ = 2.1–27.4°
*c* = 10.9761 (7) Å	µ = 2.17 mm^−^^1^
α = 76.289 (1)°	*T* = 273 K
β = 89.138 (1)°	Block, colourless
γ = 74.802 (1)°	0.50 × 0.25 × 0.25 mm
*V* = 790.73 (9) Å^3^	

### Data collection

**Table:**

Bruker SMART APEXII CCD diffractometer	3386 independent reflections
Radiation source: Rotating Anode	3261 reflections with *I* > 2σ(*I*)
Bruker HELIOS graded multilayer optics	*R*_int_ = 0.049
Detector resolution: 10.0 pixels mm^-1^	θ_max_ = 27.0°, θ_min_ = 2.8°
φ and ω scans	*h* = −9→9
Absorption correction: multi-scan (*SADABS*; Bruker, 2009)	*k* = −13→13
*T*_min_ = 0.520, *T*_max_ = 0.582	*l* = −14→13
6867 measured reflections	

### Refinement

**Table:**

Refinement on *F*^2^	Primary atom site location: structure-invariant direct methods
Least-squares matrix: full	Secondary atom site location: difference Fourier map
*R*[*F*^2^ > 2σ(*F*^2^)] = 0.022	Hydrogen site location: difference Fourier map
*wR*(*F*^2^) = 0.059	H-atom parameters constrained
*S* = 1.06	*w* = 1/[σ^2^(*F*_o_^2^) + (0.0284*P*)^2^ + 0.4222*P*] where *P* = (*F*_o_^2^ + 2*F*_c_^2^)/3
3386 reflections	(Δ/σ)_max_ < 0.001
201 parameters	Δρ_max_ = 0.71 e Å^−^^3^
0 restraints	Δρ_min_ = −0.81 e Å^−^^3^

### Special details

**Table:**

Geometry. All esds (except the esd in the dihedral angle between two l.s. planes) are estimated using the full covariance matrix. The cell esds are taken into account individually in the estimation of esds in distances, angles and torsion angles; correlations between esds in cell parameters are only used when they are defined by crystal symmetry. An approximate (isotropic) treatment of cell esds is used for estimating esds involving l.s. planes.
Refinement. Refinement of F^2^ against ALL reflections. The weighted R-factor wR and goodness of fit S are based on F^2^, conventional R-factors R are based on F, with F set to zero for negative F^2^. The threshold expression of F^2^ > 2sigma(F^2^) is used only for calculating R-factors(gt) etc. and is not relevant to the choice of reflections for refinement. R-factors based on F^2^ are statistically about twice as large as those based on F, and R- factors based on ALL data will be even larger.

### Fractional atomic coordinates and isotropic or equivalent isotropic displacement parameters (Å^2^)

**Table:**

	*x*	*y*	*z*	*U*_iso_*/*U*_eq_	
I	0.542549 (19)	0.247310 (13)	1.062688 (11)	0.02504 (6)	
F	−0.0461 (2)	0.7492 (2)	−0.01911 (13)	0.0441 (4)	
O1	0.2937 (2)	0.37876 (15)	0.50304 (13)	0.0214 (3)	
O2	0.3809 (2)	0.75140 (17)	0.66534 (15)	0.0284 (3)	
C1	0.2629 (3)	0.5691 (2)	0.57785 (18)	0.0204 (4)	
C2	0.3398 (3)	0.4484 (2)	0.67903 (19)	0.0201 (4)	
C3	0.3934 (3)	0.4255 (2)	0.80613 (18)	0.0215 (4)	
H3	0.3824	0.4982	0.8447	0.026*	
C4	0.4640 (3)	0.2880 (2)	0.87108 (18)	0.0215 (4)	
C5	0.4839 (3)	0.1760 (2)	0.8147 (2)	0.0243 (4)	
H5	0.5346	0.0861	0.8620	0.029*	
C6	0.4289 (3)	0.1976 (2)	0.68911 (19)	0.0217 (4)	
C7	0.3574 (3)	0.3354 (2)	0.62726 (18)	0.0205 (4)	
C8	0.2388 (3)	0.5210 (2)	0.47433 (19)	0.0205 (4)	
C9	0.1667 (3)	0.5850 (2)	0.34449 (19)	0.0219 (4)	
C10	0.0601 (3)	0.7217 (3)	0.3068 (2)	0.0284 (5)	
H10	0.0353	0.7757	0.3652	0.034*	
C11	−0.0100 (3)	0.7790 (3)	0.1840 (2)	0.0313 (5)	
H11	−0.0791	0.8710	0.1587	0.038*	
C12	0.0261 (3)	0.6952 (3)	0.1005 (2)	0.0298 (5)	
C13	0.1322 (3)	0.5599 (3)	0.1329 (2)	0.0303 (5)	
H13	0.1545	0.5065	0.0740	0.036*	
C14	0.2052 (3)	0.5048 (2)	0.2553 (2)	0.0259 (4)	
H14	0.2799	0.4142	0.2785	0.031*	
C15	0.4396 (4)	0.0826 (2)	0.6251 (2)	0.0295 (5)	
H15A	0.3163	0.0779	0.6072	0.044*	
H15B	0.5075	−0.0038	0.6791	0.044*	
H15C	0.5018	0.0999	0.5482	0.044*	
C16	0.0218 (3)	0.7605 (2)	0.6731 (2)	0.0279 (4)	
H16A	−0.0783	0.7464	0.6269	0.034*	
H16B	0.0467	0.6908	0.7520	0.034*	
C17	−0.0346 (4)	0.9043 (3)	0.6981 (2)	0.0383 (6)	
H17A	0.0664	0.9186	0.7417	0.057*	
H17B	−0.1411	0.9124	0.7486	0.057*	
H17C	−0.0646	0.9727	0.6198	0.057*	
S1	0.22856 (8)	0.74390 (5)	0.58266 (4)	0.02169 (11)	

### Atomic displacement parameters (Å^2^)

**Table:**

	*U*^11^	*U*^22^	*U*^33^	*U*^12^	*U*^13^	*U*^23^
I	0.03433 (10)	0.02416 (9)	0.01561 (8)	−0.00848 (6)	−0.00381 (5)	−0.00178 (5)
F	0.0453 (9)	0.0628 (11)	0.0171 (6)	−0.0101 (8)	−0.0098 (6)	−0.0001 (6)
O1	0.0293 (7)	0.0200 (7)	0.0156 (6)	−0.0069 (6)	−0.0013 (5)	−0.0048 (5)
O2	0.0352 (9)	0.0272 (8)	0.0266 (8)	−0.0114 (7)	−0.0022 (6)	−0.0101 (6)
C1	0.0250 (10)	0.0188 (9)	0.0174 (9)	−0.0055 (8)	−0.0002 (7)	−0.0046 (7)
C2	0.0234 (10)	0.0188 (9)	0.0181 (9)	−0.0056 (7)	0.0007 (7)	−0.0046 (7)
C3	0.0294 (10)	0.0200 (9)	0.0166 (9)	−0.0077 (8)	−0.0010 (7)	−0.0057 (7)
C4	0.0274 (10)	0.0222 (10)	0.0152 (8)	−0.0077 (8)	−0.0017 (7)	−0.0035 (7)
C5	0.0302 (11)	0.0197 (10)	0.0219 (10)	−0.0064 (8)	−0.0010 (8)	−0.0029 (8)
C6	0.0276 (10)	0.0193 (10)	0.0200 (9)	−0.0077 (8)	0.0017 (8)	−0.0065 (7)
C7	0.0244 (10)	0.0231 (10)	0.0158 (9)	−0.0089 (8)	0.0006 (7)	−0.0052 (7)
C8	0.0230 (10)	0.0196 (9)	0.0194 (9)	−0.0066 (8)	0.0009 (7)	−0.0042 (7)
C9	0.0245 (10)	0.0268 (10)	0.0161 (9)	−0.0107 (8)	0.0004 (7)	−0.0044 (8)
C10	0.0329 (12)	0.0287 (11)	0.0216 (10)	−0.0056 (9)	−0.0029 (8)	−0.0052 (8)
C11	0.0320 (12)	0.0311 (12)	0.0249 (11)	−0.0049 (10)	−0.0045 (9)	0.0011 (9)
C12	0.0272 (11)	0.0448 (14)	0.0164 (9)	−0.0126 (10)	−0.0034 (8)	−0.0019 (9)
C13	0.0338 (12)	0.0415 (13)	0.0197 (10)	−0.0137 (10)	0.0013 (8)	−0.0111 (9)
C14	0.0276 (11)	0.0303 (11)	0.0211 (10)	−0.0085 (9)	−0.0005 (8)	−0.0078 (8)
C15	0.0445 (13)	0.0200 (10)	0.0263 (10)	−0.0089 (9)	0.0002 (9)	−0.0092 (8)
C16	0.0291 (11)	0.0263 (11)	0.0258 (10)	−0.0035 (9)	0.0021 (8)	−0.0055 (8)
C17	0.0466 (15)	0.0275 (12)	0.0331 (12)	0.0036 (11)	0.0038 (11)	−0.0076 (10)
S1	0.0315 (3)	0.0166 (2)	0.0164 (2)	−0.00631 (19)	−0.00063 (18)	−0.00285 (17)

### Geometric parameters (Å, °)

**Table:**

I—C4	2.106 (2)	C9—C14	1.405 (3)
I—O2^i^	3.052 (2)	C10—C11	1.387 (3)
F—C12	1.357 (2)	C10—H10	0.9300
O1—C8	1.373 (2)	C11—C12	1.380 (4)
O1—C7	1.380 (2)	C11—H11	0.9300
O2—S1	1.495 (2)	C12—C13	1.376 (4)
C1—C8	1.374 (3)	C13—C14	1.387 (3)
C1—C2	1.449 (3)	C13—H13	0.9300
C1—S1	1.765 (2)	C14—H14	0.9300
C2—C7	1.389 (3)	C15—H15A	0.9600
C2—C3	1.405 (3)	C15—H15B	0.9600
C3—C4	1.391 (3)	C15—H15C	0.9600
C3—H3	0.9300	C16—C17	1.520 (3)
C4—C5	1.407 (3)	C16—S1	1.815 (2)
C5—C6	1.394 (3)	C16—H16A	0.9700
C5—H5	0.9300	C16—H16B	0.9700
C6—C7	1.385 (3)	C17—H17A	0.9600
C6—C15	1.499 (3)	C17—H17B	0.9600
C8—C9	1.465 (3)	C17—H17C	0.9600
C9—C10	1.391 (3)		
			
C4—I—O2^i^	168.75 (7)	C12—C11—H11	121.1
C8—O1—C7	106.96 (15)	C10—C11—H11	121.1
C8—C1—C2	106.76 (18)	F—C12—C13	118.6 (2)
C8—C1—S1	126.56 (16)	F—C12—C11	118.3 (2)
C2—C1—S1	126.41 (15)	C13—C12—C11	123.0 (2)
C7—C2—C3	119.10 (19)	C12—C13—C14	118.5 (2)
C7—C2—C1	105.34 (17)	C12—C13—H13	120.7
C3—C2—C1	135.56 (19)	C14—C13—H13	120.7
C4—C3—C2	116.33 (18)	C13—C14—C9	120.4 (2)
C4—C3—H3	121.8	C13—C14—H14	119.8
C2—C3—H3	121.8	C9—C14—H14	119.8
C3—C4—C5	123.00 (18)	C6—C15—H15A	109.5
C3—C4—I	117.95 (15)	C6—C15—H15B	109.5
C5—C4—I	119.06 (15)	H15A—C15—H15B	109.5
C6—C5—C4	121.11 (19)	C6—C15—H15C	109.5
C6—C5—H5	119.4	H15A—C15—H15C	109.5
C4—C5—H5	119.4	H15B—C15—H15C	109.5
C7—C6—C5	114.58 (18)	C17—C16—S1	109.14 (18)
C7—C6—C15	121.77 (18)	C17—C16—H16A	109.9
C5—C6—C15	123.63 (19)	S1—C16—H16A	109.9
O1—C7—C6	123.70 (18)	C17—C16—H16B	109.9
O1—C7—C2	110.45 (18)	S1—C16—H16B	109.9
C6—C7—C2	125.85 (18)	H16A—C16—H16B	108.3
O1—C8—C1	110.48 (17)	C16—C17—H17A	109.5
O1—C8—C9	114.21 (17)	C16—C17—H17B	109.5
C1—C8—C9	135.31 (19)	H17A—C17—H17B	109.5
C10—C9—C14	118.92 (19)	C16—C17—H17C	109.5
C10—C9—C8	122.32 (19)	H17A—C17—H17C	109.5
C14—C9—C8	118.8 (2)	H17B—C17—H17C	109.5
C11—C10—C9	121.3 (2)	O2—S1—C1	107.96 (10)
C11—C10—H10	119.4	O2—S1—C16	106.52 (10)
C9—C10—H10	119.4	C1—S1—C16	97.83 (11)
C12—C11—C10	117.9 (2)		
			
C8—C1—C2—C7	0.4 (2)	C2—C1—C8—O1	0.4 (2)
S1—C1—C2—C7	−173.92 (16)	S1—C1—C8—O1	174.74 (15)
C8—C1—C2—C3	−179.2 (2)	C2—C1—C8—C9	179.1 (2)
S1—C1—C2—C3	6.5 (4)	S1—C1—C8—C9	−6.6 (4)
C7—C2—C3—C4	0.9 (3)	O1—C8—C9—C10	162.3 (2)
C1—C2—C3—C4	−179.6 (2)	C1—C8—C9—C10	−16.3 (4)
C2—C3—C4—C5	0.6 (3)	O1—C8—C9—C14	−17.0 (3)
C2—C3—C4—I	−179.02 (15)	C1—C8—C9—C14	164.4 (2)
C3—C4—C5—C6	−1.5 (4)	C14—C9—C10—C11	0.7 (4)
I—C4—C5—C6	178.17 (17)	C8—C9—C10—C11	−178.6 (2)
C4—C5—C6—C7	0.7 (3)	C9—C10—C11—C12	1.3 (4)
C4—C5—C6—C15	−177.5 (2)	C10—C11—C12—F	178.2 (2)
C8—O1—C7—C6	−178.4 (2)	C10—C11—C12—C13	−2.0 (4)
C8—O1—C7—C2	1.4 (2)	F—C12—C13—C14	−179.7 (2)
C5—C6—C7—O1	−179.43 (19)	C11—C12—C13—C14	0.5 (4)
C15—C6—C7—O1	−1.2 (3)	C12—C13—C14—C9	1.7 (4)
C5—C6—C7—C2	0.9 (3)	C10—C9—C14—C13	−2.3 (3)
C15—C6—C7—C2	179.1 (2)	C8—C9—C14—C13	177.1 (2)
C3—C2—C7—O1	178.56 (18)	C8—C1—S1—O2	−139.72 (19)
C1—C2—C7—O1	−1.1 (2)	C2—C1—S1—O2	33.5 (2)
C3—C2—C7—C6	−1.7 (3)	C8—C1—S1—C16	110.0 (2)
C1—C2—C7—C6	178.6 (2)	C2—C1—S1—C16	−76.8 (2)
C7—O1—C8—C1	−1.1 (2)	C17—C16—S1—O2	65.53 (18)
C7—O1—C8—C9	179.96 (18)	C17—C16—S1—C1	176.96 (16)

Symmetry codes: (i) −*x*+1, −*y*+1, −*z*+2.
